# Effect of bisacodyl on rats with slow transit constipation

**DOI:** 10.1590/1414-431X20187372

**Published:** 2018-05-28

**Authors:** Yong-bing Wang, Jie Ling, Wen-zhong Zhang, Gang Li, Wei Qiu, Jun-hua Zheng, Xiao-hui Zhao

**Affiliations:** 1 Department of General Surgery, Pudong New Area People's Hospital, Shanghai University of Medicine & Health Sciences, Shanghai, China; 2 Clinical Medical School, Shanghai University of Medicine & Health Sciences, Shanghai, China

**Keywords:** Bisacodyl, Slow transit constipation, c-Kit

## Abstract

The effect of bisacodyl on the treatment of rats with slow transit constipation (STC) was studied. Forty-five female Wister rats were divided into control group, STC group, and STC bisacodyl group. The immunohistochemical method was used to determine interstitial cells of Cajal (ICC) and the expression of c-Kit protein. Body mass and the number of defecations were significantly decreased in the STC group compared with the control group on the 100th day after diphenoxylate administration, while dry weight of feces was significantly increased and the intestinal transit time was prolonged. There were significant differences in the number of defecations, dry weight of feces, and intestinal transit time among the three groups. The number of defecations was higher, dry weight of feces was lower, and intestinal transit time was shorter in the STC bisacodyl group compared to the STC group. In addition, ICC basement membrane dissolution occurred in the colon wall of the STC group. The connection between ICC and surrounding cells was destroyed, and the nucleus shrunken to different degrees. Moreover, c-Kit expression in the STC group was significantly lower than the control group. The connection between ICC and surrounding cells in the STC bisacodyl group was significantly stronger than the STC group, and the number of ICC and the expression of c-Kit were increased. Bisacodyl could reduce the severity of STC in rats by increasing the number of ICC and the expression of c-Kit.

## Introduction

Slow transit constipation (STC) is an intractable condition characterized by prolonged transit time of colonic contents that is caused by the decrease of colonic motility. STC can induce abdominal distention, stomachache, defecation difficulty, perianal diseases, and is related with cardiovascular and cerebrovascular diseases and colon cancer (1). The etiology of STC is complex and its mechanism is still not fully understood. Many studies showed that the etiology of STC is related with enteric nervous system, hormones and neurotransmitters, intestine smooth muscle, and interstitial cells of Cajal (ICC) (2,3). The ICC are pacemaker cells that produce gastrointestinal myoelectric activity and gastrointestinal motility, contributing to colon motility and fecal propulsion. Researchers have found that ICC could participate in STC. For example, Zhu et al. (4) reported that total glucosides of paeony promoted intestinal motility in STC rats through amelioration of ICC and Geramizadeh et al. found that ICC was decreased in patients with STC (5). Therefore, we focused on ICC to reveal the underlying mechanism of STC.

Bisacodyl is a common drug for the treatment of STC, which was first used in 1985. Later, researchers found that bisacodyl promoted and propagated motor activity in patients with severe STC (6). However, the mechanism of bisacodyl on ICC is not revealed and there is still a lack of adequate clinical data.

Rats have a mild disposition, good tolerance, and high success rate in experimental research, therefore they are the first choice for the study of STC (7). The STC rat model was established by continuously feeding diphenoxylate, which can be applied in clinical practice and has many advantages, such as being a simple, economical, and reproducible method (4).

In this study, we established an STC rat model and treated STC rats with bisacodyl. Our aim was to study the relationship between bisacodyl and ICC, and explore the potential underlying mechanism of STC.

## Material and Methods

### Animals

Female Wister rats, weighing 120–140 g, were purchased from Shanghai Laboratory Animal Center of Chinese Academy of Science (China), and kept in 50–70% humidity at room temperature. All procedures were approved by the Ethics Committee of the Pudong New Area People's Hospital.

### Establishment of the STC rat model

Forty-five female Wister rats were kept in separate cages for three days. Thirty rats were divided into two groups: STC group and STC bisacodyl group. The other 15 rats were used as the control group.

The rats in the STC groups were given diphenoxylate by intragastric administration (8 mg·kg^™1^·d^™1^), while rats in the control group were treated with the same amount of physiological saline orally. After 100 days of diphenoxylate treatment, rats in the STC bisacodyl group were treated with bisacodyl (20 mg·kg^™1^·d^™1^) for 30 days. The number of defecations, feces dry weight, and body weight of rats were recorded every day. One week after drug withdrawal, the rats were fasted for 24 h. The rats received 100 g/L activated carbon (2 mL) and then the time from the intragastric administration to the first black stool was recorded. The rats were caged individually and the number of defecations was measured by the metabolic cage (pellet/day). Feces were dried by natural air at room temperature and then weighed (mg/pellet).

### Measurement of c-Kit positive ICC and c-Kit expression in the colon

After one month of bisacodyl treatment, rats were anesthetized with 2% pentobarbital via intraperitoneal injection. The abdominal cavity was exposed through an abdominal median incision. Intestinal tissue (about 1 cm length) was cut from the colon 5 cm away from ileocecal valve. Then, the intestinal tissue was washed with PBS and fixed with 4% paraformaldehyde for paraffin embedding.

The sections (about 5 μm) were mounted on slides and baked on a 75°C baking instrument (Boxin Equipment lnc., China) for 10 min. Xylene solution was used for dewaxing. The procedures for hematoxylin-eosin staining were as follows: hydration for 10 s with anhydrous ethanol, 95% ethanol, and 80% ethanol; wash with water for 2 min; hematoxylin staining for 8 min; wash with flowing water for 1 min; 1% hydrochloric acid ethanol for 5 s; wash with flowing water for 20 s; 1% eosin staining for 2 min; wash with water for 2 min; dehydration of gradient ethanol for 3 min; dehydration of xylene for 5 min; and seal with neutral gum.

The procedures of c-Kit protein immunohistochemical staining were as follows: dewax and dewater the sections; soak with PBS for 5 min; sodium citrate for restoration; natural cooling for 5 min; wash with cold water for 10 min; soak with PBS for 5 min; incubate with 3% hydrogen peroxide solution for 15 min; wash with PBS for 2 min; add rabbit anti-mouse c-Kit polyclonal antibody (Thermo Scientific, USA) and incubate for 1.5 h; wash with PBS for 2 min; add enzyme labeled second antibody for 30 min; wash with PBS for 2 min; add diaminobenzidine (DAB) colored solution for 10 min; wash with distilled water; hematoxylin for counterstaining for 5 min; dehydration of gradient ethanol for 5 min; dehydration of xylene for 5 min; wash with PBS for 3 times; and seal with neutral gum.

Specimens were observed under a light microscope (×100). Blue nucleus and brown cytoplasm were determined as c-Kit-positive cells (ICC cells). Five high magnification views (×100) were randomly selected in each slice, and the number of ICC cells was calculated in each field. c-Kit expression was proportional to the area or concentration of the brown cytoplasm. The areas of five high magnification fields (×100) were measured by Image-Pro Plus 6.0, and the change of c-Kit expression is reported as the percentage of area size.

### Statistical analysis

SPSS, version 20.0, was used for data analysis (USA). Measurement data are reported as means±SD. For normally distributed data, differences between two groups were tested using Student's *t*-test, and differences among three groups were tested by one-way ANOVA. Non-normally distributed data were analyzed by non-parametric analyses, with P<0.05 considered statistically significant.

## Results

### Body mass of STC rats

**Figure 1.**
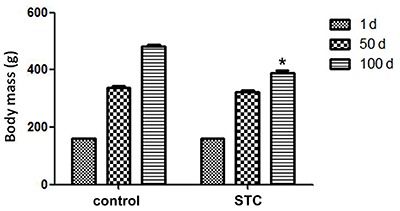
Changes of body mass in slow transit constipation (STC) rats and control rats after feeding diphenoxylate suspension. Data are reported as means±SD. *P<0.05 compared to control (ANOVA).

As shown in Figure 1, at the 50^th^ and 100^th^ day after feeding diphenoxylate suspension, body mass in STC rats was significantly lower than control rats (321.9±13.3 g *vs* 337.6±12.7 g; P=0.013 and 389.6±15.1 g *vs* 482.9±11.5 g, respectively; P=0.000). These findings indicated that the STC model was successfully established.

### Defecation condition

At the 100^th^ day after feeding diphenoxylate suspension, the number of defecations per day in the STC group was less than that of the control group (P<0.05), while dry weight of feces and intestinal transit time were greater than that of the control group (P<0.05) (Table 1).

**Table 1.** Comparison of defecation condition in control and slow transit constipation (STC) rats after 100 days of diphenoxylate treatment.

Groups	Number of defecations (pellets/d)	Dry weight of feces (mg/pellet)	Intestinal transit time (min)
Control (n=15)	41.4±5.4	145.8±21.7	408.8±64.9
STC (n=30)	24.8±5.7	193.1±15.8	515.5±100.2
*t* value	6.667	5.576	2.826
P value	0.000	0.000	0.011

Data are reported as means±SD. Between-group comparison was done with the *t*-test.

After one month of bisacodyl treatment, there were significant differences in the number of defecations, dry weight of feces, and intestinal transit time among the control group, STC group, and STC bisacodyl group (P=0.000). The number of defecations in the STC bisacodyl group was greater than that of the STC group (P<0.05), while dry weight of feces and intestinal transit time in the STC bisacodyl group were less than in the STC group (P<0.05) (Table 2).

**Table 2.** Comparison of defecation condition among control, slow transit constipation (STC) control, and STC bisacodyl groups after 1 month of diphenoxylate treatment.

Groups	Number of defecations (pellets/d)	Dry weight of feces (mg/pellet)	Intestinal transit time (min)
Control (n=15)	45.3±8.0	126.6±17.7	358.2±47.7
STC (n=15)	25.9±5.8	172.3±15.8	496.8±65.1
STC bisacodyl (n=15)	37.2±5.4*	151.1±9.3*	415.4±48.2*
*t* value	17.60	21.94	15.27
P value	0.000	0.000	0.000

Data are reported as means±SD. *P<0.05, compared with STC group (ANOVA).

### Comparison of pathological characteristics and c-Kit expression

In the STC group, the ICC basement membrane was dissolved in the colon wall. The connection between ICC and surrounding cells was destroyed, and the nucleus was shrunken to different degrees. In the STC group, the number of ICC in a single field of vision was 9.51±2.08, and c-Kit expression was (13.05±3.12%), which were significantly lower than the control group (37.12±5.36, 72.01±9.33%) (P=0.000).

**Figure 2.**
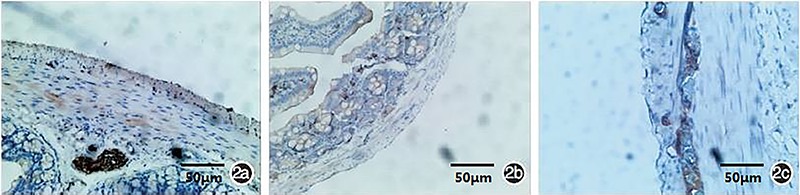
Immunohistochemical images of c-Kit protein in rat colon cells (×200, bar: 50 μm). *2a*, Slow transit constipation (STC) bisacodyl group; *2b,* STC group; *2c,* Control group.

Compared with the STC group, the connection between ICC and surrounding cells in the STC bisacodyl group was significantly strengthened, and the number of ICCs (19.04±3.51) and the expression of c-Kit (46.28±7.09%) were also increased (P=0.000) (Figure 2).

## Discussion

Constipation can severely reduce life quality of the patients and bring financial burden to the patients and their families (8,9). Recently, attention has been paid to the influence of STC on the health of patients, and further investigations are still needed to understand the pathogenesis and find an effective treatment (10,11). Our previous report showed that intestinal transit time of STC patients was significantly longer than healthy volunteers, while the frequency, contraction amplitude, and physiological responsivity of high amplitude propagated constriction (HAPC) were less than in healthy volunteers, which suggested that dynamic abnormality was a major cause of slow colonic transit and could increase constipation in STC patients (12).

Bisacodyl is a new drug that increases intestinal motility by the following possible mechanisms (13): increasing prostaglandin E2, thereby reducing colonic aquaporin 3 and promoting colonic motility, and decreasing the absorption of sodium ions, chloride ions, and moisture in colon by increasing intestinal volume. Bisacodyl or its product decomposed by intestinal bacteria can directly stimulate the intestinal wall innervation, mediate the reflex, cause intestinal reflex peristalsis, and result in defecation (14). Bisacodyl could change the migrating motor complex of colonic mobility by increase of HAPC (15). Therefore, it was speculated that long-term use of bisacodyl improves the colonic motility and ICC function.

In the current study, the 100th day, when STC rats had the most pronounced difference in body mass, was selected as the experimental time point to observe the effect of bisacodyl. The symptoms of STC rats were significantly relieved at the 30th day after feeding bisacodyl, because the number of defecations increased, dry weight of feces decreased and the intestinal transit time shortened. These findings indicated that bisacodyl improved the constipation of rats and had a good therapeutic effect.

The digestive tract, like the heart, has natural pacemakers (16). The contractile activity of the digestive tract is controlled by the slow wave of the pacemakers (17). The action potential superimposed on the slow wave potential induces contraction of smooth muscles of the digestive tract. Early in 1982, Thuneberg pointed out that ICC are the pacemaker that produce gastrointestinal myoelectric activity, which was proven by the electrophysiological test *in vitro* and *in vivo* (18). Reduction and dysfunction of ICC could result in a decrease in slow wave activity in the colon, and block information transmission between intestinal nervous system and smooth muscle cells, delaying electrical excitement transmission between pacemaker cells and smooth muscle cells, and resulting in decreased contractility of smooth muscle cells, colonic motility disorder, and fecal transit delay.

The c-Kit receptor is a transmembrane protein encoded by proto-oncogene c-kit that has tyrosine kinase activity (19). It is a marker of ICC, and can be detected by a specific antibody (20). The number of ICC in STC rats was decreased, and c-Kit expression was decreased, consistent with a previous report (5). These findings suggested that STC was related with the number of ICC and c-Kit expression. In the current study, the number of ICC and c-Kit expressions in the STC bisacodyl group were significantly increased, which indicated that the effect of bisacodyl on the treatment of STC was closely related to the number of ICC and c-Kit expressions, and provided theoretical basis for bisacodyl treatment of STC.

As a limitation, we did not observe the time for reversal of the STC model after stopping diphenoxylate treatment and there is a possibility that part of the response of bisacodyl is due to this time.
